# Polyserositis and Acute Acalculous Cholecystitis: An Uncommon Manifestation of Undiagnosed Systemic Lupus Erythematosus

**DOI:** 10.7759/cureus.4899

**Published:** 2019-06-14

**Authors:** Elena I Obreja, Carlos Salazar, Daniel G Torres

**Affiliations:** 1 Internal Medicine, Louis A. Weiss Memorial Hospital Affiliate of the University of Illinois at Chicago, Chicago, USA; 2 Rheumatology, Louis A. Weiss Memorial Hospital Affiliate of the University of Illinois at Chicago, Chicago, USA

**Keywords:** sle, acute acalculous cholecystitis, transaminitis, rare manifestation, polyserositis, lupus, autoimmunity, gall bladder, pleural effusion, pericardial effusion

## Abstract

Systemic lupus erythematosus (SLE) is a common systemic disease in the rheumatologic field. Serositis and gastrointestinal symptoms are common manifestations of SLE; however, polyserositis concurrently with acute acalculous cholecystitis is a rare and usually underestimated entity that can be associated with SLE. Medical treatment with steroids is efficacious and, in most instances, cholecystectomy can be avoided. We present the case of a young female patient with polyserositis and acute acalculous cholecystitis secondary to undiagnosed SLE, who eventually required surgical laparoscopic intervention and improved with immunosuppressive treatment.

## Introduction

The term "lupus erythematosus" was first described in the nineteenth century, and at that time, the diagnosis was limited to skin lesions. A hundred years later, the disease was found to be systemic with multiorgan involvement [1]. The most common systems involved are the skin, musculoskeletal, hematologic, renal, and neurologic. Other common manifestations are serositis and gastrointestinal (GI) symptoms, which occur in 20% and 40%, respectively [2]. The GI manifestations can occur due to the side effects of medication, vasculitis, or by autoimmune processes [3]. Acute acalculous cholecystitis (AAC) is a very rare entity that can present months or years after the diagnosis but in rare cases, it can present as the first manifestation. Based on a PubMed search, the first case of AAC as a manifestation of SLE was described in 1983. However, the first case of AAC as the first manifestation of systemic lupus erythematosus (SLE) was published in 2005 and, since then, there have been only 15 cases described. Our case encompasses polyserositis associated with rare features of this common rheumatologic disease presenting as the initial manifestation.

## Case presentation

A 22-year old Ethiopian female without a past medical history presented to the emergency department (ED) with fevers, pleuritic chest pain, flu-like symptoms, and dry cough. The patient presented with similar symptoms one week earlier. Common laboratory results and a chest X-ray (CXR) were within normal limits; therefore, she was discharged with symptomatic treatment.

However, her symptoms persisted for one week and she returned to the ED. Repeated laboratory tests were remarkable for elevated inflammatory markers, C-reactive protein (CRP), and erythrocyte sedimentation rate (ESR), and the patient was admitted for further work-up.

A review of systems revealed generalized weakness, symmetric polyarthralgia with swelling and stiffness lasting more than one hour in the morning, photosensitivity, and malaise for the past three years. The patient denied rashes, Raynaud’s phenomenon, sicca symptoms, oral ulcers, or family medical history of autoimmune disease. On physical examination, the heart was regular, with no murmurs appreciated, lungs were clear bilaterally, the abdomen was soft, non-tender, and non-distended, with no hepatosplenomegaly noticed, and the musculoskeletal exam was within normal limits.

A repeat CXR was unremarkable. The echocardiogram showed trivial pericardial effusion with no other abnormalities. Electrocardiogram (EKG) showed T wave inversions in all leads, with no ST elevations. The respiratory virus PCR panel was only positive for rhinovirus (see Table 1).

**Table 1:** Respiratory viral panel RVS: Respiratory syncytial virus

	Result	Normal value
Influenza A	Negative	Negative
Influenza B	Negative	Negative
RSV A	Negative	Negative
RSV B	Negative	Negative
Parainfluenza 1	Negative	Negative
Parainfluenza 2	Negative	Negative
Parainfluenza 3	Negative	Negative
Rhinovirus	POSITIVE	Negative
Metapneumovirus	Negative	Negative
Adenovirus	Negative	Negative

**Figure 1:**
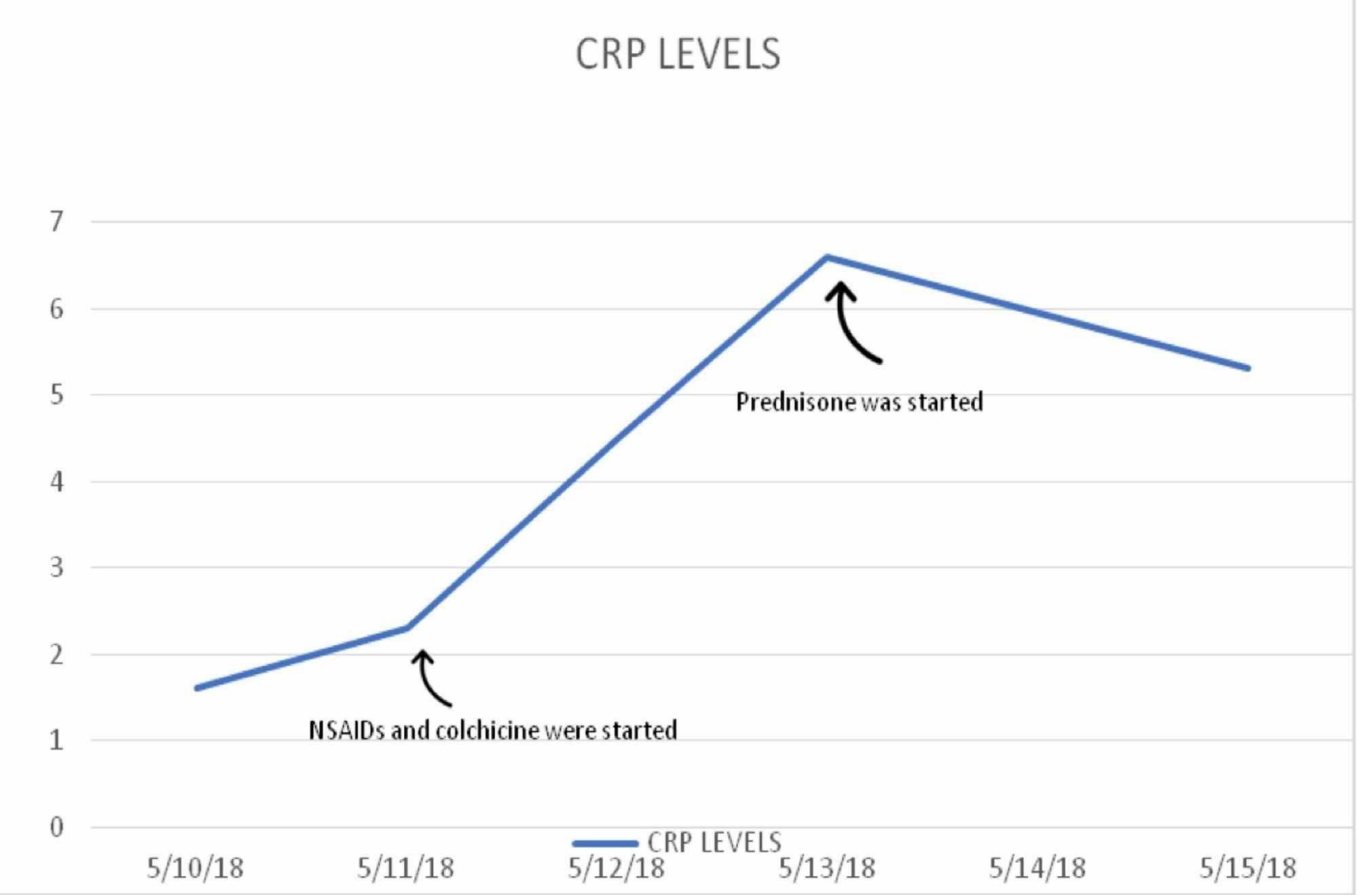
CRP levels CRP response to three days of NSAIDs and colchicine followed by prednisone CRP: C-reactive protein, NSAIDs: nonsteroidal anti-inflammatory drugs

The diagnosis of acute pericarditis was made; therefore, the patient was started on colchicine and nonsteroidal anti-inflammatory drugs (NSAIDs) with no clinical improvement. The patient reported worsening chest pain and fatigue. Her inflammatory markers were up-trending (see Figure 1). Two days later, a new right upper quadrant abdominal pain and positive Murphy’s sign complicated the patient’s hospital course.

Repeated laboratory tests showed disproportionate severe transaminitis with alanine aminotransferase (ALT) predominance, slight elevation of alkaline phosphatase, and mild hyperbilirubinemia (see Table 2 and Table 3). Hepatitis B, hepatitis C, and hepatitis A serologies were negative.

**Table 2:** Complete blood count WBC: white blood cell, RBC: red blood cell, MCV: mean corpuscular volume, MCH mean cell hemoglobin, MCHC mean corpuscular hemoglobin concentration, RDW red cell distribution width

	Results	Normal value
WBC	9.9	4.8-10.8 K/UL
RBC	4.63	4.2-5.2 M/UL
Hemoglobin	10.4	12.0-16.0 G/DL
Hematocrit	31.6	37.0-47.0 %
MCV	68	80-97 FL
MCH	22.5	27-34 PG
MCHC	32.9	30-36 G/DL
RDW	18.3	11.5-14.5 %
Platelet count	376	150-450 K/UL

**Table 3:** Comprehensive metabolic panel CO_2_: bicarbonate, ALT: alanine aminotransferase, AST: aspartate aminotransferase, GFR: glomerular filtration rate

	Results	Normal value
Sodium	136	134-148 MMOL/L
Potassium	4.4	3.5-5.5 MMOL/L
Chloride	109	95-111 MMOL/L
CO_2_	22	22-32 MMOL/L
Blood urea	8	6-21
Creatinine	0.6	0.7-1.5 MG/DL
Calcium	8.8	8.5-10.5 MG/DL
Glucose	98	70-100 MG/DL
Albumin	2.7	3.2-5.5 G/DL
Total protein	7.5	6.0-8.3 G/DL
Alk Phophatase	163	40-150 IU/L
ALT	709	6-50 IU/L
AST	631	0-40 IU/L
Bilirubin total	1.9	0.2-1.4 MG/DL
Anionic gap	5	7-14
GFR	>60	>60

**Figure 2:**
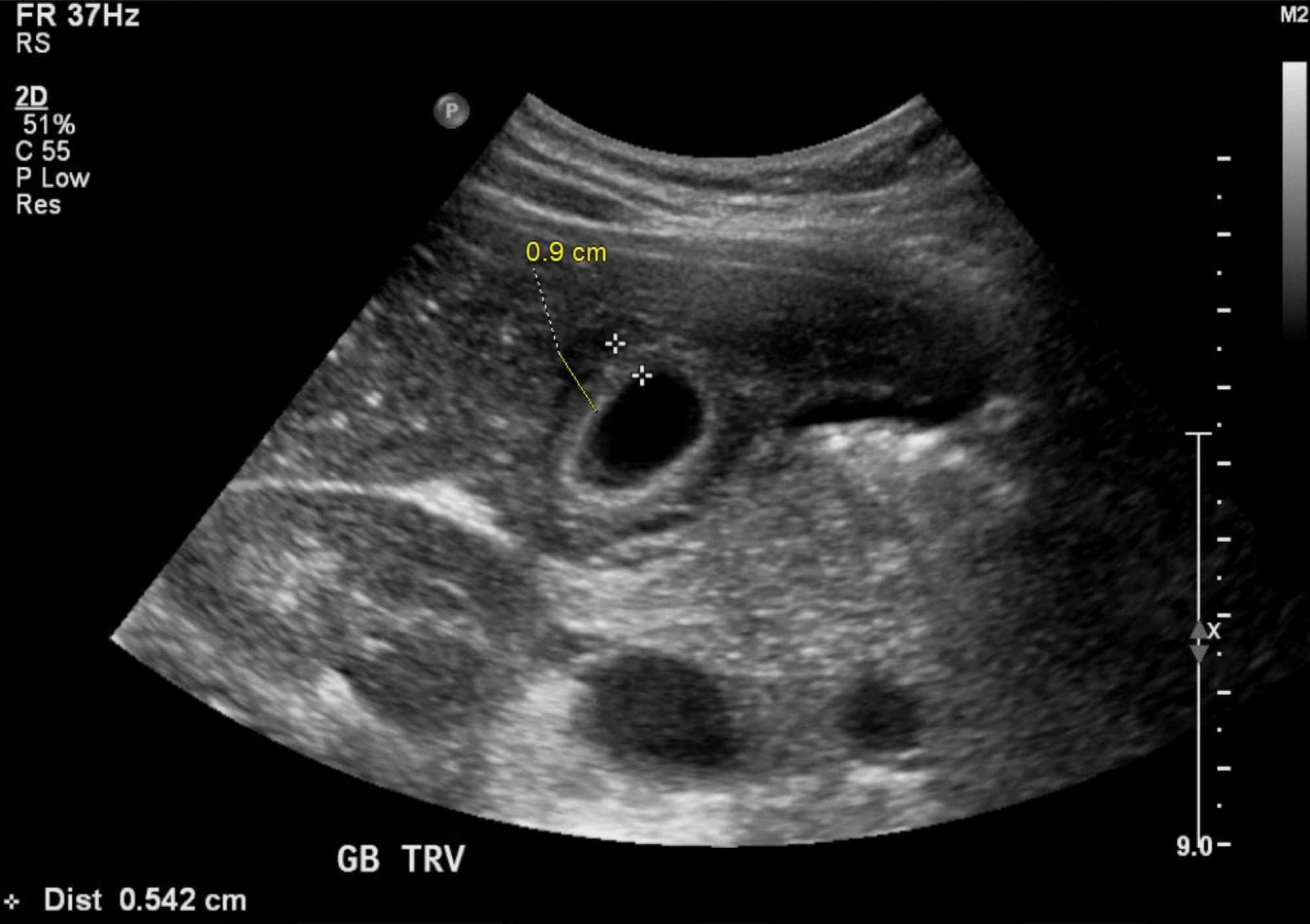
Abdominal ultrasound Abdominal ultrasound showing gallbladder dilatation more than 3 mm and pericholecystic fluid

**Figure 3:**
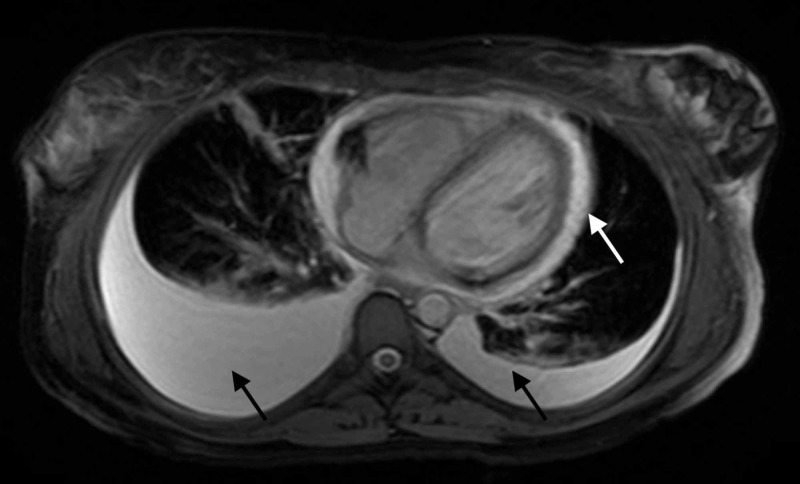
MRI of the abdomen without contrast Black arrows showing bilateral pleural effusions, moderate on the right and small on the left. White arrow showing moderate pericardial effusion.

An abdominal ultrasound showed gallbladder thickening with pericholecystic fluid, consistent with AAC (see Figure 2), confirmed by a hepatobiliary iminodiacetic acid (HIDA) scan. An MRI of the abdomen also showed AAC and revealed new bilateral pleural effusions, worsening pericardial effusion, ascites, and periportal edema (see Figure 3). Repeated echocardiography showed signs of increased pericardial effusion.

**Figure 4:**
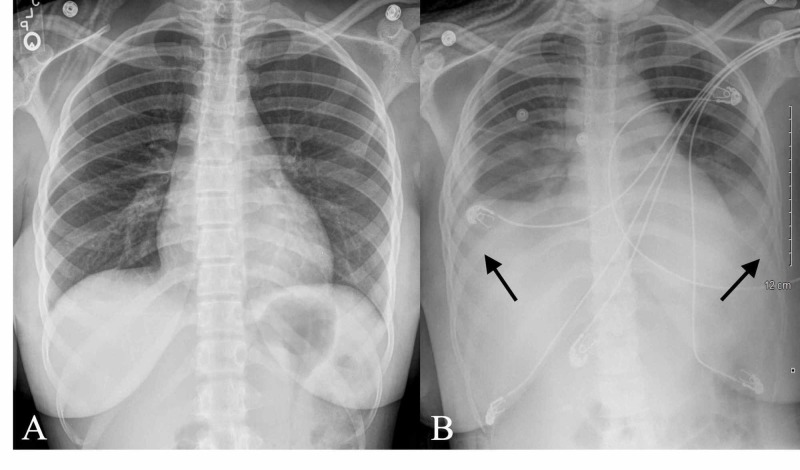
Posteroanterior and portable chest X-ray (A) Posteroanterior chest X-ray showing normal bilateral costophrenic angles and no acute intrathoracic process. (B) Portable chest X-ray on day three showing new bilateral lung base opacities. Arrows showing new bilateral pleural effusions.

Due to a rapidly evolving AAC and severe transaminitis, the surgical service was consulted, and a decision was made for the patient to undergo laparoscopic cholecystectomy. Surgical pathology of the gallbladder was consistent with acute on chronic cholecystitis without any stones or sludge noted. The patient's postoperative course was complicated by respiratory distress due to rapidly increasing bilateral pleural effusions requiring transfer to the intensive care unit (see Figure 4).

Additional autoimmune workup revealed positive anti-nuclear antibody (ANA), anti-Ro (SSA), and anti-La (SSB) antibodies, and rheumatoid factor (RF); and negative anti-CCP antibodies (ACPPA), anti-smooth muscle antibodies (ASMA), and normal complement levels (see Table 4 and Table 5). Anti-ribosomal P antibody and anti-liver kidney microsomal antibodies were not tested since the patient selectively agreed with a laboratory workup. Further imaging of wrists and hands showed non-erosive arthritis. Urine analysis was consistent with proteinuria 30 mg/dl. The patient was also found to have iron-deficiency anemia. Direct antiglobulin test (DAT) was negative.

**Table 4:** Autoimmune markers ANA: antinuclear antibodies

	Result	Reference Range
ANA	POSITIVE	Negative
Anti-RNP/SM	Negative	Negative
Anti-SSA (RO)	POSITIVE	Negative
Anti-SSB (LA)	POSITIVE	Negative
Anti-Scl 70	Negative	Negative
Anti-ds DNA	Negative	Negative
Anti-SM	Negative	Negative
Rheumatoid Factor	95	<30
Anti-CCP IGG/IGA	4	<20

**Table 5:** Inflammatory markers ESR: erythrocyte sedimentation rate

	RESULT	NORMAL VALUE
C3 Complement	160	79-152
C4 Complement	25.5	18-55
ESR	50	0-16

A diagnosis of SLE was made. Therefore, NSAIDs were stopped and the patient was started on steroids and hydroxychloroquine with a resolution of pleural and pericardial effusions, a decrease in her transaminitis, and a marked improvement of polyarthralgia.

The patient was followed up over the next several months and the steroids were slowly tapered down, with the patient eventually being treated only with hydroxychloroquine and remaining symptom-free.

## Discussion

SLE, as a prototype of autoimmunity, is a common rheumatologic disease that preferentially targets women of childbearing age. Clinicians can note the manifestations of multiple systems that are affected and pathologists can visualize the depositions of immune complexes, changes cause by inflammation, and vascular damage [4].

Two criteria guide rheumatologists in diagnosing SLE. The 1997 American College of Rheumatology (ACR) criteria and the 2012 Systemic Lupus International Collaborative Clinics (SLICC) are the two mainstay guidelines for making a diagnosis [5]. Our patient fulfilled both the ACR and SLICC criteria. The reported history of photosensitivity rash, non-erosive arthritis, pericarditis associated with bilateral pleural effusions, proteinuria, and the positive ANA made a clear diagnosis of SLE (see tables).

GI manifestations are quite common in SLE as a consequence of medication side effects and infections and usually are not caused by lupus itself [6]. GI symptoms presenting as the first manifestation of SLE is rare and misleading, causing a delay in diagnosis [7]. The GI system is affected, starting from the oral mucosa (e.g. oral ulcers) to the small and large intestine (e.g. intestinal pseudo-obstruction, protein-losing enteropathy, lupus colitis, collagenous colitis, etc.). Thrombosis of intestinal vessels can lead to lupus mesenteric vasculitis, the most common cause of GI involvement. Extra-intestinal organs are not spared. Patients can have involvement of the liver (e.g. autoimmune hepatitis, lupus hepatitis), pancreas (e.g. pancreatitis), and the biliary tract (e.g. cholecystitis) [6,8].

Our patient presented with atypical chest pain, and she was found to have pericardial and bilateral pleural effusions, which are a very common presentation of SLE. However, ascites, severe transaminitis, and acute acalculous cholecystitis are uncommon features as the initial presentation. The patient was started on prednisone 60 mg oral daily, which was slowly tapered down for one month. The patient demonstrated overall clinical improvement and resolution of her transaminitis.

Liver dysfunction, with subsequently elevated liver enzymes, occurs in 25%-59% of the patients with SLE [9]. The transaminitis seen in SLE requires the exclusion of non-autoimmune causes, such as medications, especially since many of them are hepatotoxic. Viral infections or non-alcoholic fatty liver disease (NASH) can also cause liver dysfunction. An extensive workup, including serologies, is needed; however, in many cases, a liver biopsy is done to confirm the diagnosis.

Lupus hepatitis and autoimmune hepatitis are two conditions that can affect the liver in a patient with SLE. Given the similarities in clinical and laboratory presentations, it can be difficult to differentiate them. Lupus hepatitis usually has a positive anti-ribosomal P antibody positive and distinctive features on histology. There will be evidence of mild portal infiltration with lymphocytes, neutrophils, and plasma cells and hydropic degeneration of liver cells, steatosis, mild cholestasis, focal necrosis, and nodular cirrhosis [9].

A study done by Zheng [10] confirmed that the deposit of complement C1q in the liver is strongly associated with lupus hepatitis. This study also showed that patients with higher disease activity were more prone to develop lupus hepatitis, which suggested that the occurrence of lupus hepatitis may correlate with disease activity.

Autoimmune hepatitis is still a challenging diagnosis for physicians. It is a form of chronic inflammatory liver disease with unknown etiology [11]. Systemic connective tissue diseases, such as SLE, undifferentiated connective tissue disease (UCTD), mixed connective tissue disease (MCTD), and limited scleroderma, have been associated with autoimmune hepatitis (AIH) [9]. There are three types of AIH: type I (characterized by hypergammaglobulinemia, lupus features, and positive ANA), type II (positive anti-liver-kidney microsomal antibodies with a negative ANA), and type III (positive ANA, anti-smooth muscle antibody, and anti-liver pancreas antigen). Histologically, it is characterized by periportal piecemeal necrosis and hepatocyte rosette formation with plasma cell and lymphocyte infiltrate [9].

A study done by Matsumoto et al. [12] of 160 patients with different collagen diseases (SLE, rheumatoid arthritis, mixed connective tissue disease, dermatomyositis, polymyositis, polyarteritis nodosa, and scleroderma) showed that only three patients (two with SLE and one with MCTD) were found to have histologically proven AIH. However, the other three patients who had clinical, serological, and biochemical data positive for AIH had negative histologic findings for AIH.

Other features that can distinguish these two entities are the prognosis and progression of the disease. Autoimmune hepatitis progresses to cirrhosis and has a five-year survival of 25% for untreated patients and 80% for treated patients versus lupus hepatitis, which has benign progression and a good prognosis [9].

Our patient was found to have severe transaminitis with alanine aminotransferase (ALT) and aspartate aminotransferase (AST) ranging in their 700s mg/dl and hyperbilirubinemia, which can be explained by acute acalculous cholecystitis. However, in newly diagnosed SLE who presents with polyserositis, a positive ANA, and a negative anti-smooth muscle antibody (ASMA), lupus hepatitis cannot be fully excluded. Even though a liver biopsy was not done, the dramatic response to steroids with the normalization of liver enzymes and benign progression kept lupus hepatitis higher in the differential diagnosis. The patient's hepatic function was monitored in the outpatient setting, and it remained within normal limits.

Acute acalculous cholecystitis is known to have a high mortality and morbidity rate, and it usually presents in hospitalized and critically ill patients [13]. There is a male preponderance and it has also been reported after surgical interventions, such as open abdominal aortic reconstruction and cardiac surgery, and in patients who have undergone bone marrow transplantation [13].

Peritonitis and ascites are rare manifestations in a lupus flare [3]. Abdominal serositis is part of the ACR criteria for diagnosing SLE. 

A chart review done by Al-Hakeem et al. [14] showed that out of 88 patients diagnosed with SLE monitored over a 15-year period, 15% presented with abdominal pain. The diagnosis accounting for the abdominal pain in these cases was cholecystitis in only one patient who also underwent for cholecystectomy.

Another study done by Yang H et al. [15] showed that only 0.15% of patients with SLE were identified to have AAC out of which 30% of the cases were presenting as the first manifestation. All of the patients were treated with steroids and did not require surgical intervention.

The diagnosis of AAC is made by abdominal ultrasound, which will show a significance for gallbladder wall thickening more than 3 cm and pericholecystic fluid without any gallstones identified [16]. In most of the cases, steroids are the first choice of treatment.

AAC is a life-threatening condition, and it usually occurs in critically ill patients. In very rare occasions, it can also be seen in patients with SLE as a lupus flare or as a first manifestation of it.

## Conclusions

Our case encompasses two common entities in patients with systemic lupus erythematosus: pericarditis and massive bilateral pleural effusions. Interestingly enough, our patient also had associated gastrointestinal and hepatic manifestations. AAC is extremely rare as the first manifestation of SLE, and it usually has a poor prognosis when not associated with connective tissue disease. Severe transaminitis can be seen in the setting of AAC; however, it could not exclude the diagnosis of lupus hepatitis given the immediate favorable response to steroids. The constellation of these conditions occurred as the first manifestation of SLE, with rapidly evolving symptoms.

Having a good physical examination along with a detailed history can provide us with clues for accurate diagnosis followed by better treatment. There have been several cases of autoimmune diseases with uncommon characteristics of connective tissue diseases as the first manifestation. In this setting, most of the time, the diagnosis can be delayed or sometimes even missed, hence, patients do not receive appropriate treatment.

Therefore, our case represents the typical illustration of an early rapidly evolving systemic lupus erythematosus associated with rare autoimmune features.
